# Presence of the Human Cytomegalovirus in Glioblastomas—A Systematic Review

**DOI:** 10.3390/cancers13205051

**Published:** 2021-10-09

**Authors:** Inti Peredo-Harvey, Afsar Rahbar, Cecilia Söderberg-Nauclér

**Affiliations:** 1 Department of Neurosurgery, Karolinska University Hospital, 171 76 Stockholm, Sweden; inti.peredo-harvey@sll.se; 2 Department of Medicine, Solna, BioClinicum, Karolinska Institutet, 171 64 Stockholm, Sweden; afsar.rahbar@ki.se; 3 Department of Neurology, Karolinska University Hospital, 171 76 Stockholm, Sweden

**Keywords:** glioblastoma, human cytomegalovirus, immunohistochemistry, in situ hybridization, polymerase chain reaction

## Abstract

**Simple Summary:**

Whether the human cytomegalovirus (HCMV) is present in samples obtained from patients with glioblastoma (GBM) has been a matter under debate during the last two decades. Many investigators have demonstrated the presence of HCMV proteins and nucleic acids in GBM tumors, while some have not been able to detect it. It is important to evaluate current data and resolve these issues to clarify the possible role of the HCMV in GBM tumorigenesis and if this virus can serve as a potential target of therapy for these patients. In the present systematic review, we aim to review published research studies with a focus to identify differences and similarities in methods used for the detection of the HCMV in GBM samples found to be positive or negative for HCMV. Our data suggest that the HCMV is highly prevalent in glioblastomas and that optimized immunohistochemistry techniques are required to detect it.

**Abstract:**

Glioblastoma is a malignant brain tumor with a dismal prognosis. The standard treatment has not changed in the past 15 years as clinical trials of new treatment protocols have failed. A high prevalence of the human cytomegalovirus (HCMV) in glioblastomas was first reported in 2002. The virus was found only in the tumor and not in the surrounding healthy brain tissue. Many groups have confirmed the presence of the HCMV in glioblastomas, but others could not. To resolve this discrepancy, we systematically reviewed 645 articles identified in different databases. Of these, 81 studies included results from 247 analyses of 9444 clinical samples (7024 tumor samples and 2420 blood samples) by different techniques, and 81 articles included 191 studies that identified the HCMV in 2529 tumor samples (36% of all tumor samples). HCMV proteins were often detected, whereas HCMV nucleic acids were not reliably detected by PCR methods. Optimized immunohistochemical techniques identified the virus in 1391 (84,2%) of 1653 samples. These data suggest that the HCMV is highly prevalent in glioblastomas and that optimized immunohistochemistry techniques are required to detect it.

## 1. Introduction

Glioblastoma is a highly malignant brain tumor. With an annual incidence of 2–3 new cases per 100,000 individuals, glioblastomas are the most common primary brain tumor. The prognosis is dismal. After surgery and combined radio- and chemotherapy, the expected 5-year survival rates are less than 5% and less than 10% for tumors expressing wild-type and mutant isocitrate dehydrogenase, respectively [1]. Unfortunately, advances in knowledge of glioblastomas over the last decades have not led to new effective treatments. This discouraging situation suggests that the causes and pathogenesis of glioblastomas remain unknown.

In 2002, Cobbs and his research group reported that the human cytomegalovirus (HCMV) was highly prevalent in glioblastoma samples, but was not present in the surrounding healthy brain tissue [2]. These findings were not confirmed by all available studies, and thus the notion that the HCMV is prevalent in glioblastomas became controversial. In a 2011 consensus statement about HCMV detection in glioblastomas [3], a group of oncologists and virologists described the detection of HCMV proteins and HCMV nucleic acids in these tumors, and discussed the need to optimize both sample preparation and detection techniques for appropriate HCMV diagnostics.

In 2014, scientists from Sweden and Finland used an automated immunohistochemical (IHC) staining procedure with optimized staining protocols to compare 9 commercial HCMV antibodies in 544 glioblastoma samples from 68 patients. The virus was detected in 90% of the samples. The HCMV immediate-early (IE) protein, pp65 protein, and early and late proteins were present in nuclear and cytoplasmic cellular compartments [4]. Although this large study identified the HCMV in most of the samples, certain antibodies did not perform well in detecting certain HCMV proteins, despite the use of optimized techniques. In a meta-analysis of 32 studies that included 2190 samples of all types of glioma (low-grade astrocytoma, glioma, and glioblastoma), the overall estimated HCMV prevalence was 63% [5]. However, many investigators have reported that they were unable to detect the HCMV in glioblastomas.

HCMV is a β-herpes virus found in 80–100% of healthy adults in various populations [6]. Like other herpes viruses, the HCMV is never eliminated from its host after a primary infection. Instead, it establishes a persistent asymptomatic latent infection [7] that can be reactivated during an individual’s life. HCMV has the largest genome of all herpes viruses. It was initially believed to encode 165 proteins [8] from 165–252 open reading frames (ORFs); however, ribosomal profiling detected 751 unique RNAs in infected cells [9], suggesting that HCMV is much more complex than previously thought. The HCMV also encodes 4 long noncoding RNAs and 26 miRNAs that affect human cell biology. This complexity reflects the long co-evolution of the HCMV and its host, during which HCMV mechanisms affecting cell and immunological functions emerged to help the virus co-exist with its host. These mechanisms must be considered when investigating the role of the HCMV in cancer.

The HCMV is considered to be oncomodulary rather than oncogenic. Through sophisticated strategies, it can alter the malignant properties of cells; however, most viral strains do not initiate cellular transformation [3,10,11]. Although all HCMV strains can establish all the hallmarks of cancer [12,13,14], certain strains may also have oncogenic properties [12,15,16,17] and possibly be more frequently associated with various forms of cancer. HCMV gene products can modulate glioblastoma proliferation and angiogenesis, and confer resistance to apoptosis and the ability to evade host immune strategies. Thus, conventional treatment against the virus may improve the patient´s response to chemotherapy or immunotherapy, and thereby improve the prognosis, as our team has proposed [18,19].

In view of the controversy surrounding the prevalence of HCMV nucleic acids and proteins in glioblastomas, we systematically reviewed the literature on HCMV diagnostics in glioblastoma. Our goals were to synthesize current knowledge and to ascertain why some investigators were able to detect the HCMV in glioblastomas, while others could not.

## 2. Materials and Methods

**Figure 1:**
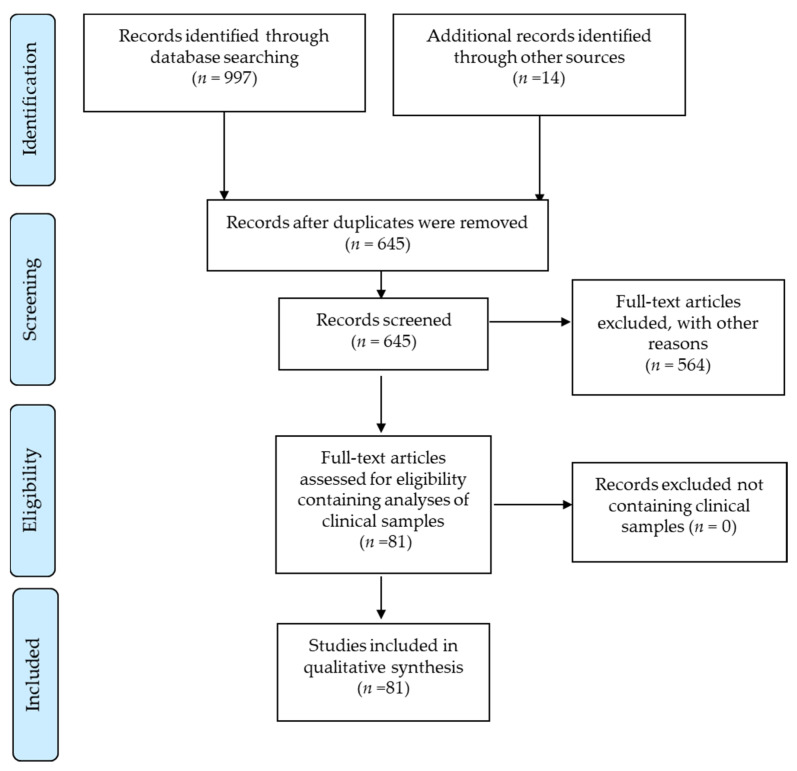
PRISMA 2009 Flow Diagram (from Mober D, Liberti A, Tetzlaff J, and Altman DG; The PRISMA Group (2009). PreFigure 6. e 1000097 (access until 31 August 2021). For more information, visit WWW.prisma-Statement.org (access until 31 August 2021). Other sources appear as indicated.

From a search in MEDLINE (Ovid, access until 31 August 2021), Embase (Embase.com, access until 31 August 2021), and Web of Science until August 31, 2021, and in Google Scholar from 2018 to 2021, we identified and reviewed 997 published articles, exclusive of duplicates (Figure 1).

The search terms were glioblastoma; gliosarcoma; glioma; glioblastoma; gliosarcoma or glioma in combination with cytomegalovirus; human cytomegalovirus or human herpesvirus 5; salivary gland virus; and HCMV or HHV-5 in humans. Those terms were used to search for the title, abstract, keyword, or heading words of the articles in the indicated databases, and identified 631 articles (Figure 1 and Figure S1). We also identified 14 articles from other sources (sent to us by colleagues or identified by a Google search) that were published after 2 July 2018, which included analyses of clinical samples for HCMV (Prisma Flow diagram in Figure 1). From these 645 articles, we selected for in-depth review those that included analyses of clinical samples from glioblastoma patients (*n* = 81).

### 2.1. Analyses of Sample Preparation and Identification of Methods Used for HCMV Analyses

For each article, we evaluated how the clinical samples were handled and prepared for the analyses, specifically, of which method was used for HCMV testing. We recorded the number of samples analyzed in each study; how many different analyses were conducted in each article; which methods were used for HCMV testing; and whether the result was positive, negative, or inconclusive.

Some articles clearly described techniques and methods used for HCMV analyses, while others did not. Therefore, in the tables, the results are presented by the technique used to identify the virus in tumor or blood samples, even if methodological details were sparse. For tumor samples, immunohistochemical (IHC) staining, in situ hybridization (ISH), immunofluorescence (IF), polymerase chain reaction (PCR), flow cytometry analysis (FACS), and next-generation sequencing (NGS) were used for HCMV analyses. For blood samples, PCR, enzyme-linked immune assay (ELISA, to detect HCMV-IgG and IgM), and FACS (HCMV antigen T-cell stimulation tests) were used to identify HCMV proteins in blood cells or in plasma to determine whether the patient had developed an antibody response to HCMV (IgG or IgM analyses) or a T-cell response to HCMV peptides. In some studies, samples were listed as IgG/IgM positive; the results are summarized as such in the tables.

IHC staining analyses of tumor samples was conducted with antibodies for different HCMV proteins: immediate-early (IE) 1, IE2, early (EA), or late proteins (LA). Some studies used antibodies to immediate-early antigen (IEA) without specifying whether IE1 or IE2 antigen expression was analyzed; the results are specified as such in the tables. For each method used, the results are presented as the percentage of samples positive for the HCMV in each analysis and the total number of samples tested is indicated.

### 2.2. Identifying Optimal Methods for More Accurate HCMV Testing

To ascertain why some investigators were able to detect the HCMV in glioblastomas, while others could not, we analyzed the methods used for HCMV testing in depth when samples were highly positive versus negative for HCMV. IHC and PCR analyses gave divergent results, while NGS was uniformly negative for the HCMV. ISH analyses for HCMV nucleic acids had the highest prevalence of HCMV. For IHC analyses of HCMV prevalence, we further analyzed how tumor samples were prepared; which fixation methods were used; whether postfixation/antigen retrieval protocols were used; and which antibodies were used for the HCMV testing of samples with high prevalence, low prevalence, or negative results. For different PCR methods, we recorded which primers were used and whether any special protocol was used for sample preparation.

## 3. Results

Our database search identified 631 articles with search terms for HCMV in glioblastoma, gliosarcoma, or glioma patients (hereafter referred to as glioblastoma patients). Fourteen additional articles from other sources were also included, as they contained analyses of the HCMV in clinical samples from the same time period under analysis. From these 645 articles, we selected those that had examined clinical samples for in-depth analyses (Figure 1). In 81 of these articles (Table S1), 9444 tumor samples from 3770 glioblastoma patients and 2420 blood samples from 1561 glioblastoma patients were tested for HCMV in, respectively, 190 analyses of tumor samples and 57 analyses of blood samples (Table 1).

HCMV was detected in glioblastoma samples in 51 of these articles [2,4,19,20,21,22,23,24,25,26,27,28,29,30,31,32,33,34,35,36,37,38,39,40,41,42,43,44,45,46,47,48,49,50,51,52,53,54,55,56,57,58,59,60,61,62,63,64,65,66,67], while 30 studies found no evidence of the HCMV [68,69,70,71,72,73,74,75,76,77,78,79,80,81,82,83,84,85,86,87,88,89,90,91,92,93,94,95,96,97] (Table 1 and Table S1). Table 2 summarizes studies that did or did not identify the virus in GBM tumor specimens and in blood samples, the number of samples tested, and the method used for the analyses. Evidence of the HCMV was found in 2529 (36.0%) of 7024 tumor samples analyzed using IHC, ISH, IF, or PCR methods. Evidence of the HCMV was found in 1094 (45.2%) of 2420 blood samples analyzed using ELISA, FACS, or PCR techniques.

### 3.1. HCMV Protein Detection in Tumor Specimens

The technique most frequently used to detect HCMV proteins was IHC, which was used in 74 analyses with 3111 (44.3%) of 7024 tumor samples in 39 articles. In most of these studies, the antibodies were for HCMV IE1 or IE2 proteins (regulatory proteins acting as transcription factors), HCMV tegument proteins (pp65, pp71, and pp28 in the viral particle), HCMV late antigens, and HCMV p52/76-kDa or p43/76-kDa proteins. IHC identified HCMV proteins in 1500 (21.4%) of the 7024 tumor samples in 51 (68.9%) of 74 analyses. IF was used to detect viral proteins in 217 (3.1%) of 7024 tumor samples and, of those 217 samples, 119 (54.8%) showed presence of HCMV proteins (Table 2).

### 3.2. HCMV IE Protein Detection

Antibodies to HCMV IE1 (68–72-kDa protein) were used on 773 tumor samples from 21 studies. Samples were positive for HCMV IE1 in 14 studies [2,4,26,27,29,36,38,46,50,56,57,63,87] and negative in 7 [44,67,69,76,82,85,96]. In 389 of 773 samples (50.3%), HCMV IE1 proteins were detected with antibody clones E13 [87], 6F8.2 [4], or 8B1.2 [4,26,46]; in 13 studies, the clones were not specified [2,27,29,36,38,50,56,57,63,67,69,82,85]. In 3 studies, all 73 tumor samples analyzed with HCMV IE2 antibodies were positive [40,41,43] (Table 3). In 7 studies, IEA antibodies (not specifying IE1 or IE2) identified the virus in 70.3% of samples (230 of 327) [2,19,52,54,59,70,95]. In 6 of these studies, the HCMV prevalence was 97.5% (230 of 236 samples) [2,19,52,54,59,95]. In the remaining study, however, the antibody clone MCA2147 did not detect HCMV IEA in any of the 91 samples analyzed [70].

The HCMV early antigen (EA) was analyzed in 159 tumor samples in 3 analyses in 2 studies [4,85]. Evidence of an EA viral protein was found in 122 (76.7%) samples (Table 3). One clone, 8B1.2 [4], gave positive staining results in 61 (89,7%) of 68 tumor samples (Table 4), whereas in 1 of 2 studies [4,85] the antibody clone QB1/42 was positive (Table 4) and the other study found no evidence of HCMV (Table 5). The study that demonstrated the presence of HCMV was done with optimized techniques [4].

The HCMV pp65 protein was analyzed in 18 studies [2,4,27,29,36,38,46,47,50,57,63,66,69,70,76,78,85,95]. In 11 of these studies [4,29,36,38,46,47,63,66,76,78,85], the HCMV pp65 protein was analyzed with antibody clones 2 and 6, which detect the C-terminus of pp65 in 630/3111 (20.3%) of the IHC samples (Table 4). The HCMV pp65 protein was found in 329 (52.2%) of 630 tumor samples. The antibody clones were from Abcam (3 positive), Novocastra (2 negative, 2 positive), Leica (3 positive), and Vector (1 negative). In 8 of these studies, 329 (67.7%) of 486 samples were positive [4,29,36,38,46,47,63,66]; 3 studies found no evidence of the HCMV pp65 protein using the same antibody clone [76,78,85]. It is important to note that two negative studies used higher dilutions of antibodies [76,85] (1:800 and 1:200), while one negative study used a lower dilution (1:50). The 1:200 dilution was used on thicker tissue sections (8 μm) [85] than the 6 μm that is recommended for analyzing the HCMV in tumor specimens. The studies that detected the HCMV pp65 proteins with clones 2 and 6 used antibody dilutions of 1:40, 1:50, and 1:200 on 6 μm tissue sections, or 1:50 and 1:75 dilutions on 4 μm sections [29,46]. In 2 studies, a monoclonal antibody identified the HCMV pp65 protein in 40 (93.0%) of 43 samples [2,50]. In 3 studies [27,69,95] that did not mention the clone used or its origin, the HCMV pp65 protein was found in 22 (48.9%) of 45 tumor samples. In 1 study, the antibody clone 12D10 identified the HCMV pp65 protein in 28 (77.8%) of 36 tumor samples [57]. One study [70] used the antibody clone 26 of unknown origin and showed no virus in 91 tumor samples using tissue sections with 3 μm thickness (Table 5).

The detection of the HCMV in tissue samples requires optimized immunohistochemical techniques with antibodies capable of showing the presence of the virus in tumor tissues. Summarized information about the antibodies that worked well for the detection of HCMV protein expression in GBM tissue specimens are shown in Table 4. The virus was identified in 1391 (84.2%) of 1653 samples in 37 studies (Table 4).

The antibody cocktail CCH2 + DDG9 was used to detect IE and E antigens in eight studies [4,24,70,73,78,88,89,92]. In 7 studies, analysis of 379 tumor specimens with different antibody concentrations found no evidence of the HCMV. In 1 study, HCMV proteins were detected in 40 (25.3%) of 158 samples; the dilution was not specified [24].

Thus, the HCMV was not detected with clones 26 or an unspecified monoclonal antibody to the HCMV pp65 protein, and the antibody cocktail CCH2 + DDG9 in eight of nine studies using QB1/42 was negative (Table 5).

### 3.3. HCMV Late Protein Detection

The antibodies to HCMV late antigen (LA) were used in six studies [4,19,52,54,57]. In 5 studies of 369 tumor samples [4,19,52,54,57], 275 (74.5%) were positive (Table 3). In 2 studies with the clone 1G5.2 at a dilution of 1:100 [57] and 1:400 [4], 87 (83.7%) of 104 samples were positive (Table 4). In 3 studies [19,52,54] with an unspecified clone, 188 (95.4%) of 197 samples were positive (Table 4). In 1 study, all 68 samples analyzed with the antibody clone 2D4.2 to late antigen [4] were negative (Table 5).

### 3.4. Optimized Techniques More Often Reveal Viral Proteins in Glioblastomas

To understand why some investigators found HCMV proteins in a high percentage of glioblastoma samples whereas others found no evidence of HCMV proteins, we investigated which methods were used when a positive versus a negative result was obtained. Although details of staining protocols were not always specified, in 37 studies, antibodies showed HCMV protein expression in 84.2% of tumor specimens (Table 4); antibodies for early or late proteins or the HCMV pp65 protein in these same studies showed a lower prevalence of HCMV expression. However, in seven of eight studies in which the CCH2 + DDG9 antibody cocktail was used, no evidence of HCMV IE and early proteins was found (Table 5). Furthermore, the clone MCA2147 to HCMV IE (used in only 1 study) did not find HCMV in 91 tumor samples [70]; the clone 26 to the HCMV pp65 protein was also negative in these samples [70] (and may represent clones 2 and 6, referred to by others). Negative results were obtained with clones 2 and 6 to the HCMV pp65 protein in three of the eleven studies [76,78,85]. The clone QB1/42 to an HCMV EA antigen was negative in 0 of 23 samples [85] and positive in 61 of 68 tumor samples [4]. The clone 2D4.2 to a late HCMV antigen were negative in all of 144 tumor samples examined, respectively [4,44]. Lower dilutions of antibodies were used in studies with positive results than in those with negative results.

Baumgarten et al. [70]—who did not find evidence of HCMV proteins using four different antibodies to HCMV IE, the HCMV pp65 protein, and early protein—did not use an antigen retrieval protocol that is thought to be important to detect HCMV in tumor specimens [2,4,98]. Likewise, Polterman [85] used clone QB1/42 without an optimized protocol for HCMV protein detection and found no evidence of the HCMV. HCMV protein detection can be optimized by using methods for antigen retrieval, such as using enzyme treatment with pepsin (1.25 mg/mL for 3 min at 37 °C) in combination with a preheated buffer in microwave and then in water bath and heat treatment at 50 °C for 2 h or, alternatively, enzyme treatment with pepsin (1.25 mg/mL) for 1 min at 37 °C followed by heat-induced epitope retrieval in a pressure cooker for 15 min [2,54,99,100,101]. Optimized enzyme treatment with pepsin (20 μg/mL and 15 min at 37 °C) in combination with heat treatment in a water bath at 50 °C for 2 h [2,50,54] or heating samples in a pressure cooker without enzyme treatment are critical for antigen retrieval. The pH of the antigen retrieval buffer should be the same as specified in the product sheet for each antibody (usually 7.5–9.0).

These optimization steps were first described by Cobbs’s research team [2] and variants of these optimized protocols were later described by other investigators, including our own team, as essential for detecting HCMV proteins in glioblastoma specimens [3,4,50,98,102]. Libard et al. [4] used an automated protocol (Dako Autostainer Plus, DakoCytomation, Copenhagen, Denmark) and reported that optimization was important; however, no details were provided. In that study, HCMV IE was detected with clone 8B1.2 or 6F8.2, the HCMV pp65 protein with clone 2/6, early protein with clone BM204, and late protein with clone IG5.2 in 90% of 68 patients [4]. The HCMV IE protein clone QB1.42 and the late protein clone 2D4.2 were not detected; and no evidence of HCMV was found with the CCH2 + DDG9 antibody cocktail, despite the use of optimized techniques for HCMV protein detection. Sabatier et al. found HCMV DNA in 7 of 9 glioblastoma samples, but detected HCMV IE proteins with clone E13 in only 9 of 81 samples [87]. These investigators did not explain whether an optimized staining protocol was used. However, they used older glioblastoma samples (from 1980–2003), which may have negatively influenced the staining results. They also used Bouin’s fluid fixative, which is less sensitive when immunoperoxidase staining protocols are used on tissue specimens. Moreover, Bouin’s fluid fixative contains picric acid, which can degrade DNA and RNA, and thereby compromise the detection of intact DNA with certain methods, such as ISH and PCR [102].

Another important factor seems to be the thickness of the tissue sections used for IHC staining. In studies in which section thickness was specified, those that found a high prevalence of HCMV proteins had used sections of 6 μm [4]. Lau, Zavala, Bassett, and Yang et al. did not mention section thickness [67,69,78,96], and Polterman et. al., who found no evidence of the HCMV, analyzed 8 μm sections [85]. Thus, we conclude that optimized protocols for HCMV protein detection in glioblastoma tissue specimens are essential for IHC staining results that indicate a high prevalence of the HCMV.

Certain antibodies (CCH2/DDG9, 8B1.2, MCA2147, Q1/42, 2D4.2, 26, and unoptimized use of clones 2.6 and MAB810) (Table 5) did not perform well for the detection of HCMV proteins in tumor specimens. Samples examined for HCMV protein expression with these antibodies and no optimization protocols for HCMV protein detection represented 34,8% of all samples. If these samples are excluded (for having used unsuitable methods), HCMV proteins were detected in 1391 of 1653 (84.2%) glioblastoma samples analyzed with different antibodies and optimized techniques.

### 3.5. Detection of HCMV Nucleic Acids in Tumor Samples

To detect HCMV DNA or RNA, PCR methods were used in 48 studies of 2676 tumor samples (38.1% of all 7024 tumor tissue samples) that included 85 analyses [2,20,21,22,23,24,25,26,27,28,29,30,31,33,37,38,41,42,44,47,48,50,51,55,57,60,61,62,67,68,70,72,73,74,75,76,78,81,82,83,84,85,86,88,90,92,95,96]. ISH was used in 15 studies of 339 tumor samples that included 18 analyses [2,36,44,50,52,54,56,61,63,66,76,78,87,95,96]. NGS was conducted in 681 tumor samples in 7 studies [71,72,77,91,93,94,97].

#### 3.5.1. Detection of HCMV Nucleic Acids by PCR Methods

The primers most commonly used for PCR methods were for HCMV gB, IE, or pp65 genes, but investigators also used primers to pp71, US28, UL144, and other genes for HCMV DNA or RNA detection. PCR revealed evidence of HCMV DNA or RNA in 778 of 2676 samples (29.1%) (Table 6). Nested PCR was used to analyze 604 (22.6%) of 2676 samples and detected HCMV genome in 151 (25.0%). Quantitative/real-time PCR was used to analyze 1817 (67.9%) of 2676 samples and found evidence of the HCMV in 489 (26.9%). In 3 studies in which reverse transcriptase was used to make cDNA for PCR template, HCMV RNA was detected in 48 (53.9%) of 89 samples. These studies used primers for US28 (56.7% positive) [55,60] or pp71 (90.0% positive) [48,55]. One study used ddPCR and found no evidence of the HCMV [81]. One study used a set of 20 HCMV-specific primers for PCR amplification and found evidence of the HCMV in 140 of 144 samples (97.2%) [55]. Another study used PCR-based amplification of 46 HCMV gene regions; sequencing analysis confirmed the presence of HCMV DNA in 17 of 17 samples [25]. Five cancer-associated HCMV genotypes, which were different from strains of congenitally infected infants, were segregated by pp65 variants, implying that unique HCMV strains may be associated with glioblastomas [25].

HCMV pp65 primers were used to analyze 641 (24.0%) of 2676 tumor samples and detected HCMV pp65 in 185 samples (28.9%). In 637 (23.8%) of the 2676 samples, gB primers detected HCMV gB in 183 samples (28.7%). In 260 of the samples (9.7%), IE-specific primers detected HCMV IE in 72 samples (27.7%). In 126 of the samples (4.7%), UL144 primers were used and identified the viral genome in only 8 samples (6.3%). In 60 of the samples (2.2%), US28-specific primers were used and found HCMV US28 in 34 samples (56.7%). In 30 of the samples (1.1%), HCMV pp71 primers were used and found HCMV in 27 (90%). In 654 of the samples (24.4%), undefined PCR primers were used and showed the HCMV genome in 150 (22.9%). In 268 of the samples (10.0%), other primers to the HCMV (UL17, UL27, UL69, UL96, US2, US11, US17, UL112, and UL73) identified HCMV nucleic acids in 119 (44.4%) samples (Table 6). Few samples were analyzed in each study; primers to UL17, UL27, UL69, UL96, and US11 found the virus in 100% of samples, but only 100 samples were analyzed with these primers. Only one study was negative and used primers to US17 [76].

#### 3.5.2. Detection of the HCMV Genome by Next-Generation Sequencing

In 7 studies, NGS was used to detect the HCMV in 681 tumors. DNA was sequenced in two of these studies [71,94]; RNA was sequenced in four [77,91,93,97]; and one was a metagenomic analysis [72]. None of these studies found evidence of HCMV nucleic acids (Table 7).

#### 3.5.3. Detection of the HCMV Genome by ISH

In 18 analyzes performed in 15 studies, ISH was used [2,36,44,50,52,54,56,61,63,66,76,78,87,95,96] to analyze 339 tumor samples for HCMV DNA or RNA. The HCMV was detected in 132 samples (38.9%) (Table 7). The HCMV prevalence was high in 10 analyses (130 of 152 samples, 85.5%) [2,36,50,52,54,56,63,66,87] and lower in eight (2 of 187 samples, 1.1%) [44,61,76,78,95,96]. Positive results were obtained with probes to early RNA or DNA, IE1, or pp65 DNA, or total HCMV DNA. Negative results were obtained with probes to early RNA, pp65 DNA, or pp150 DNA (Table 7).

### 3.6. Analyses of Blood Samples to Detect HCMV DNA, HCMV-Specific Antibodies, or HCMV-Reactive T-Cells to HCMV

Blood samples from 1561 patients with glioblastoma were analyzed using PCR methods (883 samples) [24,26,27,29,30,31,50,53,76,79,80,82,84,85,86], flow cytometry (288 samples) [32,36,45,49,53,64], and serology tests (1249 samples) [22,24,27,30,34,35,37,38,48,51,53,58,62,65,70,72,76,82,86,88].

#### 3.6.1. PCR Analyses

In 15 articles, 883 blood samples were analyzed for HCMV (20 analyses) DNA or RNA samples (Table 8). PCR analyses—defined as RT, TaqMan, and quantitative—in 453 (51.3%) of 883 samples detected the virus in 55 (12.1%) (Table 8). Nested PCR was used for 79 of 883 (8.9%) samples and the HCMV was found in 15 (19.0%). Undefined PCR methods were used on 332 (37.6%) samples and detected the virus in 168 (50.6%) samples (Table 8).

#### 3.6.2. HCMV Serology Analyses

HCMV serology tests were performed in 20 studies of 1249 samples.

As expected, all studies showed that a majority of glioblastoma patients had been infected with HCMV. Of 883 serum samples tested, 562 (63.6%) were positive for HCMV IgG (Table 2). HCMV-IgG prevalence ranged from 27.3% to 100%. The mean prevalence was 66.8% and the median was 66.9%, which is consistent with the expected HCMV prevalence in the general population. Of 297 samples tested for HCMV IgM, 33 (11.1%) were positive. In two studies, the prevalence of HCMV IgG or IgM was not specified [70,88]. In 1 of these studies, 43 (64.2%) of 67 samples were positive for HCMV IgG/IgM [70]; the other study reported only 2 pediatric GBM patients (among 21 patients with other brain tumors) and found HCMV IgG/IgM in both [88] (Table 9). These observations suggest an acute or a reactivated infection in a higher proportion of glioblastoma patients than would be expected for the general population (usually 1–3%) [103,104,105,106].

#### 3.6.3. Detection of HCMV-Reactive T cells

The reactivity of T cells to HCMV IE and pp65 peptides in blood cells has, to our awareness, been evaluated in 8 analyzes in 6 studies of glioblastoma patients, which showed reactivity to either viral peptide in 216 of the 288 samples analyzed (75%) [32,36,45,49,53,64]. In 1 of the studies [53], T-cells from 185 (73.1%) of 253 patients and from 10 (83.3%) of 12 patients with negative serology [53] were activated by HCMV IE or pp65 peptides (Table 9). Serology tests on the same patient cohort revealed 69% positivity for HCMV IgG (29 of 42) and 16.7% positivity for HCMV IgM (7 of 42 patients). Age- and gender-matched controls had a similar HCMV IgG prevalence, but only 3% were IgM positive. Tumor specimens from all patients were positive for HCMV protein. In another study [36], T cells from all 11 patients (100%) tested reacted to both HCMV IE and pp65; the tumors were positive for HCMV IE protein in 10 of the patients and for HCMV pp65 (clone 2.6) in five [36]. T cells from 265 tested samples reacted to HCMV pp65 in 195 cases (73.6%) [32,45,49,64]. Thus, the immune reactivity of T cells to HCMV peptides is very high in glioblastoma patients, possibly including a substantial proportion of those who are seronegative for the HCMV, but whose tumors are HCMV positive.

### 3.7. Detection of HCMV Proteins Using Western Blot Analyses

In eight articles [25,40,41,43,48,59,95,96], the presence of HCMV proteins was analyzed using the Western blot technique. In 5 of these studies [25,41,43,48,95], tissue specimens from 81 tumor samples were analyzed, of which 71 (87.7%) showed the presence of virus. Three studies did not mention details regarding the number of analyzed samples, but reported evidence of HCMV IE86 and pp28 proteins in the samples [40,59,66].

## 4. Additional Finding

We found three case rapports showing presence of the HCMV in glioblastomas [107,108,109]. Three articles [18,110,111] were relevant for the treatment of the HCMV in glioblastoma patients, but none of them presented results of the HCMV detection in samples from patients.

One meta-analysis study showed evidence of HCMV infection in patients with glioma [5], based on 32 studies with 2190 specimens analyzed using different laboratory techniques. In this study, the overall estimated frequency of the HCMV in glioblastomas was 62% and the most prevalent viral markers were IE1 antigen (83%), pp65 protein (62%), pp65 nucleic acids (61%), and gB nucleic acids (39%). The results of this meta-analysis support our conclusions that the HCMV is present in glioblastoma samples.

Another systematic review [112] of selected articles aimed to answer the relationship between the HCMV and GBMs. This analysis was performed based on a mathematical analysis. The authors concluded that HCMV infection is the cause of GBMs. Mathematical analyses from this study provided strict significant evidence of the cause–effect relationship between the HCMV and GBMs.

Two studies [37,62] and one case report [107] detected HCMV reactivation during radiotherapy with clinical implications that suggests the need for antiviral treatment.

One study of 68 GBM patients [113] was conducted to analyze the potential risk factors in the pathogenesis of glioblastoma. The application of the logistic regression model showed that, among others, a previous infection with HCMV was (in a statistically significant manner) associated with a glioblastoma risk.

## 5. Discussion

Since 2002, when Cobbs et al. reported 100% prevalence of the HCMV in glioblastomas, many studies have detected HCMV nucleic acids and proteins in these tumors, but others have not. Our review of 645 published articles identified 51 articles that found the HCMV in the majority of glioblastoma specimens and 30 that did not (as concluded by the authors). Our analysis suggests that the primary reason for this discrepancy relates to technical issues. Various methods were used to identify the HCMV in tumor specimens, including IHC, IF, PCR, ISH, and NGS. Serology or T-cell assays were used to determine immune reactivity to the HCMV in blood samples of glioblastoma patients. Each of these methods consistently yielded similar results by different investigators. Several factors emerged as important for optimized staining protocols, including tissue section thickness, fixation, post-fixation, pretreatment of samples (with proteinase K, heat, or microwave), the use of correct pH for respective antibody, and incubation time. The antibody clone used also affected whether the results were positive or negative. Thus, the method used is the critical determinant of whether HCMV proteins can be detected in glioblastoma.

The importance of technical issues concerning HCMV detection in glioblastoma was highlighted by Cobbs [114] in his response to a study by Baumgarten et al. [70], who found no evidence of the HCMV in 123 glioblastoma samples by IHC and PCR methods. According to Cobbs the failure to find the HCMV reflected the shortcomings of their immunostaining protocol, such as the elimination of critical technical procedures. Libard’s study also stressed the importance of optimizing the staining protocol [4] for HCMV detection. In another study [50], three methods were used in parallel to analyze 162 glioblastomas: IHC, ISH, and PCR. Optimized antigen-retrieval techniques detected the HCMV in >90% of the samples: 93% were positive for IE1 and 91% were positive for pp65. ISH detected nucleic acid sequences of the IE gene in 100% of samples. The importance of using optimized techniques for HCMV protein detection by IHC was also discussed in a consensus statement from several independent investigators experienced in HCMV diagnostics in glioblastomas [3,4,101,114].

It has also been suggested that the optimized antigen-retrieval protocols and high antibody concentration required to detect HCMV protein in brain tissue by IHC may give false-positive results [92]. Others have argued that optimization is not needed to detect other viruses in cancer specimens and implied that optimized staining protocols could lead to false-positive results [89,92]. However, in several studies that reported positive IHC results, appropriate positive and negative controls were included in the staining assays and did not show inappropriate staining results [2,4,52,54]. In our own research, IHC and ISH data show a similar consistently high prevalence of the HCMV in glioblastoma tissue specimens (close to 100%). Therefore, we conclude that optimized staining protocols are required to detect the HCMV in cancer specimens. The reason for this remains unknown.

Our analysis also revealed that some antibodies are a poor choice for detecting HCMV proteins. For example, the antibody cocktail CCH2/DDG9 failed to detect HCMV proteins in seven of eight published studies [4,24,70,73,78,88,89,92]. We too, were unable to detect the HCMV with this cocktail, despite using optimized techniques for paraffin embedded tissue specimens (unpublished observations). The single study that showed positive results with this antibody cocktail identified HCMV proteins in only 25.3% of samples [24]. In 8 analyses of 537 samples by IHC—accounting for 17.3% of such analyses—only 40 (7.4%) were HCMV positive. According to Dako, the epitope recognized by CCH2 is heat and pH sensitive, but even optimization did not, in our or other studies, yield positive results. Instead, the data suggest that the epitopes recognized by these antibodies are sensitive to formalin fixation and paraffin embedding or that they are not expressed in glioblastomas.

Antibodies might perform poorly for several reasons. Clinical isolates of the HCMV contain multiple mutations [25,115,116] and the HCMV strains detected in glioblastomas are more closely related to the Merlin strain than to AD169. Such differences may affect epitope recognition by certain antibody clones. Differences in gene mutations or deletions in the HCMV strains may also affect the detection of the encoded proteins with certain antibodies, as was the case with the CCH2/DDG9 antibody cocktail. The epitopes recognized by these antibodies might not be present or the proteins might not be expressed in HCMV-infected glioblastomas. It is also possible that the HCMV strains associated with glioblastomas have mutations in pp65. Likewise, mutations or gene deletions would also affect the ability to detect HCMV DNA or RNA with PCR methods.

Therefore, a critical question that arises is concerned with determining whether certain HCMV strains are associated with glioblastomas. A recently isolated HCMV strain, HCMV-DB, induced tumor transformation in normal mammary epithelial cells in vitro and gave rise to fast-growing triple-negative breast cancers in a mouse model [16]. Other HCMV strains did not have these effects. Similar results were reported in the 1970s, when a clinical isolate from a patient induced cellular transformation of normal human cells and gave rise to tumors in a mouse model [15]. Most other HCMV strains do not have such transforming abilities. It is therefore possible that certain HCMV strains are oncogenic and that some strains preferentially infect the brain; these may be rare and glioblastomas are indeed not prevalent tumors.

The method used to detect the HCMV is clearly a critical determinant of the results. However, it is unclear why optimized methods are required to detect HCMV proteins in glioblastomas, when parallel samples from HCMV-infected AIDS patients, used as positive controls in the same studies and protocols, are positive for the HCMV. Likewise, PCR or sequencing methods consistently performed poorly in detecting HCMV nucleic acids. In contrast, ISH methods that use a probe for HCMV DNA, and not a polymerase-based assay, more often detected HCMV nucleic acids in glioblastomas. We also noted that PCR assays using cDNA as template, after conversion of HCMV RNA with reverse transcriptase, were consistently better at detecting HCMV nucleic acids. Some primers also seemed to be better suited to detect HCMV DNA. For example, US28 gave the highest prevalence [48,55,60], and UL144 and US17 the lowest prevalence, for HCMV DNA detection [96]. Thus, assays that aim to read the code of viral DNA from a tumor specimen are consistently problematic for the detection of HCMV. Furthermore, in 7 studies of 681 glioblastomas, NGS failed to detect HCMV DNA or RNA; these studies accounted for 9.7% of all tumor samples and 89% of those analyzed for RNA. NGS also relies on polymerases to create sequencing libraries, which may help to explain the uniformly negative results. Interestingly, NGS identified Epstein–Barr viral DNA, but not HCMV DNA [71].

NGS sequencing lacks the sensitivity to detect small amounts of DNA. Since viral DNA may be present in a minority of glioblastoma cells [55], NGS may not detect small amounts of HCMV DNA/RNA in glioblastoma specimens. However, a more likely explanation is that PCR-based protocols lack specificity to identify HCMV DNA. The problem may be technical, as ISH detects the virus in most tumor cells. Viral nucleic acids and proteins may be affected by the high oxidative stress and the acidic and hypoxic environment in tumor cells, limiting their detection by conventional techniques designed to identify viral nucleic acids and proteins produced by infected non-tumor cells. Together, these findings favor the hypothesis that the HCMV strains associated with glioblastomas, unlike other strains, can replicate or at least express viral proteins in tumor cells.

Several questions emerge from these observations. HCMV does not replicate in tumor cells infected in vitro. If HCMV does replicate in tumor cells in vivo, how does it replicate in cells that are not halted in the G1 phase of the cell cycle, which is required for HCMV replication in non-tumor cells? How does the intracellular environment affect the nucleotides, protein structure, or the ability of HCMV proteins to bind to other proteins that may mask their presence? And, most importantly, do certain HCMV strains have yet undefined features associated with glioblastomas?

Is it possible that the HCMV is not present in glioblastomas and that optimized techniques to detect it yield artefactual signals? Perhaps this is the case, given the poor detection of the virus by PCR methods and the consistently negative results of NGS. Why, then, would ISH show a high prevalence of the HCMV, often matching data obtained by optimized IHC staining techniques? In our research, optimized ISH and IHC staining techniques detect the HCMV in almost all tumor specimens, whereas most PCR assays we used detect the HCMV in only a minority of samples, and exhaustive efforts to sequence the virus from glioblastoma specimens have failed. In a majority of glioblastoma patients, T cells are highly reactive to HCMV IE and pp65 peptides, consistent with the results of optimized IHC staining and ISH. Therefore, other explanations must be searched for to clarify why this virus is difficult to identify by PCR and sequencing methods in tumor tissue specimens.

Many other key questions remain unanswered. Why is the HCMV difficult to detect in tumor specimens with conventional techniques? Why do some patients with HCMV protein-positive tumors not develop antibodies to the virus? If the virus is indeed present in glioblastomas, how active is it? Is an active HCMV infection an epiphenomenon unrelated to tumor biology, or does it affect the initiation or progression of glioblastomas and, thus, have a clinical relevance? Is the activity of the virus low or is it merely difficult to measure the activity of the tumor-associated virus? These questions cannot be answered until problems with HCMV detection have been resolved.

There are indeed many problems with the detection of the HCMV in tumor specimens. Although such problems can be used to cast doubt on the potential relevance of the HCMV in glioblastomas, it seems clear that something unusual is going on with HCMV in these tumors. In biology, important insights often come from investigating unexpected and unexplainable phenomena, which merit efforts to understand them. In a retrospective study of 102 glioblastoma patients treated with valganciclovir, we found that the 2-year survival rate was 49.8% compared with 17.8% in 231 control patients receiving the same base line therapy [18]. If optimal therapy was used (surgery, temozolomide, and radiation), the 2-year survival rate was 63.9% versus 27.6% and the median overall survival was 29.7 months versus 17 months in matched controls (*p* < 0.001) [18]. Several randomized clinical phase II studies are ongoing to determine whether antiviral treatment for the HCMV or boosting the immune response to the HCMV (DC vaccination, adoptive T cell therapy, or vaccines) in glioblastoma patients prolongs their survival. The outcomes of these studies will help to reveal whether the HCMV has a role in the progression of glioblastomas.

## 6. Conclusions

In this systematic review, we found that the HCMV was present in 39.4% of 3012 blood and tumor samples obtained from GBM patients. However, several factors proved to be of key importance in explaining discrepancies in the detection of HCMV protein in glioblastomas. When optimized techniques were used, 84.2% of tumor samples were positive for HCMV proteins. HCMV DNA was often detected with ISH probes, whereas HCMV nucleic acids were not reliably detected by polymerase-based techniques, and NGS sequencing failed to detect the HCMV. Additional studies are needed to understand the mechanisms underlying these phenomena. Nevertheless, HCMV appears to be highly prevalent in glioblastomas. Further studies are needed to determine why optimized methods are required to detect the virus and why it is difficult to detect viral nucleic acids by PCR and sequencing techniques. More importantly, we need to clarify whether HCMV affects the biology, development, or progression of glioblastomas, and whether antiviral treatments or immunotherapies directed against the HCMV are useful therapeutic strategies for patients with these devastating tumors.

## Figures and Tables

**Table 1:** Total number of reviewed studies, patients, and results regarding the detection of HCMV in tissue specimens and blood samples obtained from GBM patients.

		Number of Patients	
		(*n* = 3770)	(*n* = 1561)
	Evidence of HCMV (%)	HCMV in Tissue Specimens (%)	HCMV in Blood Samples (%)
Articles (*n* = 81)	51/81 (63.0)		-
Analyses (*n* * = 247)	192/247 (77.7)	141/190 (74.2)	51/57 (89.5)
Samples (*n* º = 9444)	3623/9444 (38.4)	2529/7024 (36.0)	1094/2420 (45.2)

*n*: numbers; *n* *: number of analyses performed in the articles; *n* º: number of samples analyzed in the articles; HCMV: human cytomegalovirus.

**Table 2:** Summarized results reported from published studies with or without detection of the HCMV in clinical samples obtained from GBM patients using IF, IHC, ISH, PCR, ELISA, NGS and FACS methods.

Methods	Tumor Tissue Specimens				Blood Samples			
	IF	IHC	ISH	PCR	NGS	FACS	PCR	ELISA
	(%)	(%)	(%)	(%)	(%)	(%)	(%)	(%)
Articles	4/4	25/39	10/15	29/48	0/7	6/6	8/15	14/20
	(100)	(64.1)	(66.7)	(60.4)	0	(100)	(53.3)	(70.0)
Analyses	6/6	51/74	11/18	73/85	0/7	8/8	14/20	28/29
	(100)	(68.9)	(61.1)	(85.9)	0	(100)	−70	(96.6)
Positive	119/217	1500/3111	132/339	778/2676	0/681	216/288	238/883	640/1249
Samples	(54.8)	(48.2)	(38.9)	(29.1)	0	(75.0)	(27.0)	(51.2)
Analyzed	217/7024	3111/7024	339/7024	2676/7024	681/7024	288/2420	883/2420	1249/2420
Samples/	(3.1)	(44.3)	(4.8)	(38.1)	(9.7)	(11.9)	(36.5)	(51.6)
Total number of samples								

ISH: in situ hybridization; PCR: polymerase chain reaction; NGS: next-generation sequencing; IF: immunofluorescence; IHC: immunohistochemical staining; FACS: flow cytometry analysis.

**Table 3:** Summarized results from published studies showing expression of the HCMV proteins in GBM tissue specimens using different antibodies specifically targeting HCMV-proteins by immunohistochemical staining.

Methods	IE1 *	IE2 **	IEA	EA	LA£	pp65	CCH2 + DDG9	Other
	(%)	(%)	(%)	(%)	(%)	(%)	(%)	(%)
Expression of the HCMV	389/773	73/73	230/327	122/159	275/533	329 ^∞^/630 ^∞^	40/537	42/79
	50.3	100	70.3	76.7	51.6	52.2	7.4	53.2
Analyzed Samples/Total samples (n = 3111)	773/3111	73/3111	327/3111	159/3111	533/3111	630/3111	537/3111	79/3111
	(24.9)	(2.4)	(10.5)	(5.1)	(17.1)	(20.3)	(17.3)	(2.5)

IE: immediate early; LA: late antigen; EA: early antigen; IEA: immediate early antigen; *: 68–72 kDa; **: 86 kDa; £: 47–55kDa; £: clone CCH2 + DDG9; *n*: numbers; ∞: includes two electron microscopy analyses. Other antibodies used include pp28, US28, and Clone CMV01.

**Table 4:** Detection of the HCMV expression in GBM tissue specimens using different antibodies targeting HCMV proteins by immunohistochemical staining.

Antibodies Targeting the HCMV	Total Number of Analysis (*n* = 37)	HCMV-Positive Samples (%) (*n* = 1391/1653 (84,2)	References
IE72 (*n* = 368)	10	256/368 (69.6)	[2,27,29,36,50,56,63,87]
IE1 clone 8B1.2 (*n* = 68) (optimized methods)	1	61/68 (89.7)	[4]
IE1 clone 6F8.2 (*n* = 68)	1	61/68 (89.7)	[4]
IE2 (*n* = 73)	3	73/73 (100)	[40,41,43]
IEA (*n* = 236)	6	230/236 (97.5)	[2,19,52,54,59,95]
EA clone QB1/42 EA clone BM204 (*n* = 136)	2	122/136 (89.7)	[4]
LA (*n* = 1 97)	3	188/197 (95.4)	[19,52,54]
LA clone1G5.2 (*n* = 104)	2	87/104 (83.7)	[4,57]
pp65 (*n* = 93)	4	79/93 (84.9)	[2,27,50,57]
pp65 clone 12D10 (*n* = 36)	1	28/36 (77.8)	[57]
pp65 clones 2 and 6 (*n* = 274)	6	206/274 (75.2)	[4,29,38,46,47,66]

*n*: number of samples.

**Table 5:** No detection of the HCMV expression in GBM tissue specimens using different antibodies targeting HCMV proteins by immunohistochemical staining.

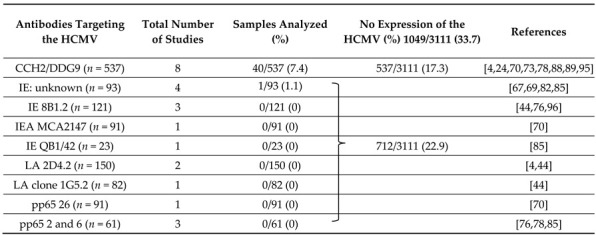

*n*: number of samples; IE: immediate early; LA: late; PP: polypeptides.

**Table 6:** Detection of the HCMV genome in GBM tissue specimens using PCR methods.

*HCMV* Genes	No Def	Nested	RT, Taq, q	RT	ddPCR	Samples	%	%/tot 2676	Analysis
	Pos/tot	Pos/tot	Pos/tot	Pos/tot	Pos/tot	Pos/tot			
No def	13/22	31/146	106/441		0/45	150/654	22.9	24.4	19
gB	5/13	84/258	94/366			183/637	28.7	23.8	19
pp71	5/5		22/25			27/30	90	1.1	4
IE1		26/73	21/162	25/25		72/260	27.7	9.7	12
pp65	6/18	10/11	169/612			185/641	28.9	24.0	9
US28	8/8		8/8	18/44		34/60	56.7	2.2	3
UL144	4/5	0/116	4/5			8/126	6.3	4.7	3
Others	49/50		65/198	5/20		119/268	44.4	10.0	16
Samples	90/121	151/604	489/1817	48/89	0/45	778/2676	29.1		
%	74.4	25	26.9	53.9	0	29.1			
%/tot 2676	4.5	22.6	67.9	3.3	1.7			100	
Analysis	14	20	47	3	1				85

PCR: polymerase chain reaction; RT: reverse transcriptase; qPCR: quantitative PCR; Taq: TaqMan PCR; ddPCR: droplet digital PCR; gB: glycoprotein B; PP: polypeptide; US: unique short; UL: unique long; tot: total; %: percent; No def: not defined. Other primers to HCMV include UL17, UL27, UL69, UL96, US2, US11, US17, UL112, and UL73.

**Table 7:** Summarized results from 7 published studies for the detection of HCMV DNA and transcripts using NGS, metagenomic analysis, and ISH techniques.

Methods	NGS DNA	NGS RNA	Meta RNA	ISH RNA	ISH DNA
Samples	0/55	0/606	0/20	10/117	122/222
Analysis	2	4	1	5	13

ISH: in situ hybridization; NGS: Next-Generation Sequencing.

**Table 8:** Summarized results from published studies for the detection of the HCMV genome in blood samples obtained from GBM patients using PCR methods.

HCMV Genes	No def	Nested	RT, Taq, q	RT	Samples	%	%/tot 883	Analysis
	Pos/tot	Pos/tot	Pos/tot	Pos/tot	Pos/tot			4
No def	6/41			0/19	6/60	10	6.8	7
gB	27/40	15/56	0/27		42/123	34,1	13.9	5
IE1	135/251	0/23	18/149		153/423	36,2	47.9	3
pp65			33/239		33/239	13,8	27.1	1
US17			4/38		4/38	10,5	4.3	
Samples	168/332	15/79	55/453	0/19	238/883	27		
%	50.6	19	12.1	0	27.0			
%/tot 883	37.6	9	51.3	2.2			100	
Analysis	6	4	8	2				20

PCR: polymerase chain reaction; RT: reverse transcriptase; qPCR: quantitative PCR; Taq: TaqMan PCR; ddPCR: droplet digital PCR; gB: glycoprotein B; PP: polypeptide; US: unique short; tot: total; %: percent; No def: not defined.

**Table 9:** Summarized results from published studies on HCMV serology using ELISA and T-cell reactivity against HCMV peptides using FACS in GBM patients.

Methods	HCMV-IgG	HCMV-IgM	HCMV-Ig	HCMV-IE Peptides (T Cell Stimulation)	HCMV-pp65 Peptides (T Cell Stimulation)
Analyses	12/12	5/7	2/2	2/2	3/3
Samples (%)	562/883 (63.6)	33/297 (11.1)	45/69 (65.2)	21/23 (91.3)	195/265 (73.6)

## Supplementary Materials

The following are available online at https://www.mdpi.com/article/10.3390/cancers13205051/s1, Figure S1: Illustration of the use of Boolean operators as support tools for the refinement process of the data search in MEDLINE (Ovid), Embase (Embase.com), and Web of Science until August 2021, and in Google Scholar from 2018 to 2021, Table S1: Information about all 81 analyzed articles.
