# Supplementary material for: Presence of the Human Cytomegalovirus in Glioblastomas—A Systematic Review
Source: Cancers (Basel). 2021 Oct 9;13(20):5051. doi: 10.3390/cancers13205051 (PMC8533734; doi:10.3390/cancers13205051)
Supplement: Supplementary file 1 [file cancers-13-05051-s001.zip › cancers-1320877-supplementary.pdf]

# Supplementary Materials:Presence of the Human Cytomegalovirus in Glioblastomas—A systematic review

**Inti Peredo-Harvey, Afsar Rahbar and Cecilia Söderberg-Nauclér**

| | |
|---|---|
| Interface: Ovid<br><br>Date of Search: 31 August 2021<br><br>Number of hits: 356<br><br>Comment: In Ovid, two or more words are automatically searched as phrases; i.e. no quotation marks are needed | Field labels<br><br>• exp/ = exploded MeSH term<br>• / = non exploded MeSH term<br>• .ti,ab,kf. = title, abstract and author keywords<br>• adjx = adjacent within x words, regardless of order<br>• * = truncation of word for alternate endings |
| 1. Glioblastoma/<br>2. Gliosarcoma/<br>3. Glioma/<br>4. (glioblastom* or gliosarcom* or gliom*).ti,ab,kf.<br>5. or/1-4<br><br>6. Cytomegalovirus/<br>7. (human cytomegalovirus or human herpesvirus 5 or salivary gland virus* or CMV or HCMV or HHV-5).ti,ab,kf.<br>8. or/6-7<br><br>9. 5 and 8<br><br>10. 9 not (animals not humans).sh. | |
| Interface: embase.com<br><br>Date of Search: 31 August 2021<br><br>Number of hits: 268<br><br>Comment: Emtree is the controlled vocabulary in Embase | Field labels<br><br>• /exp = exploded Emtree term<br>• /de = non exploded Emtree term<br>• ti,ab = title and abstract<br>• NEAR/x = adjacent within x words, regardless of order<br>• * = truncation of word for alternate endings |
| #1 'glioblastoma'/de<br>#2 'gliosarcoma'/de<br>#3 'glioma'/de | |

#4 glioblastom*:ti,ab,kw OR gliosarcom*ti,ab,kw OR gliom*ti,ab,kw
#5 #1 OR #2 OR #3 OR #4
#6 'human cytomegalovirus'/exp
#7 'human cytomegalovirus':ti,ab,kw OR 'human herpesvirus 5':ti,ab,kw OR 'salivary gland virus*':ti,ab,kw OR hcmv:ti,ab,kw OR 'hhv 5':ti,ab,kw
#8 #6 OR #7
#9 #5 AND #8
#10 #9 NOT ([animals]/lim NOT [humans]/lim)

Interface: Clarivate Analytics

Date of Search: 31 August 2021

Number of hits: 353

Field labels

- TS/Topic = title, abstract, author keywords and Keywords Plus
- NEAR/x = adjacent within x words, regardless of order
- * = truncation of word for alternate endings

TOPIC: ((glioblastom* or gliosarcom* or gliom*))

AND

TOPIC: ((human cytomegalovirus or human herpesvirus 5 or salivary gland virus* or HCMV or HHV-5))

Interface: Google Scholar

Date of Search: 2018-2021

Number of hits: 51

Search: glioblastoma|gliosarcoma|glioma "human cytomegalovirus"|HCMV

**Figure S1.** Illustration of the use of Boolean operators as support tools for the refinement process of the data search in MEDLINE (Ovid), Embase (Embase.com), and Web of Science until August 2021 and Google Scholar 2018 to 2021.

**Table S1.** Information about all 81 analysed articles.

| # | Author | Year | Ref. No | Pos/No Pos | Positive Results | *n* = | Used Techniques for Detection of HCMV |
|---|---|---|---|---|---|---|---|
| 1 | Wrensch et al. | 2001 | 65 | P | 37 | 56 | ELISA IgG |
| 2 | Coobs et al. | 2002 | 2 | P | 22 | 22 | IHC IE1-72 (only GMB) |
| | | | | | 8 | 8 | IHC EA |
| | | | | | 10 | 10 | IHC pp65 |
| | | | | | 2 | 2 | Immunogold staining pp65 |
| | | | | | 8 | 8 | ISH early gene RNA |
| | | | | | 4 | 4 | ISH total DNA |
| | | | | | 7 | 9 | Nested-PCR gB |
| 3 | Lau et al. | 2005 | 78 | N | 0 | 8 | IHC pp65 |

| | | | | | | | |
|---|---|---|---|---|---|---|---|
| | | | | | 0 | 8 | IHC CCH2/PPG9 |
| | | | | | 0 | 8 | ISH early gene (RNA) |
| | | | | | 0 | 8 | ISH total DNA |
| | | | | | 0 | 8 | PCR gB DNA |
| 4 | Sabatier et al. | 2005 | 87 | N | 9 | 81 | IHC IE1-72 |
| | | | | | 7 | 9 | ISH HCMV DNA |
| 5 | Lehrer et al. | 2011 | 79 | N | 0 | 5 | Reverse Transcription PCR blood |
| 6 | Poltermann et al. | 2006 | 85 | N | 0 | 23 | IHC IE1 68-72 |
| | | | | | 0 | 23 | IHC EA |
| | | | | | 0 | 23 | IHC pp65 |
| | | | | | 0 | 24 | Nested PCR IE1 |
| | | | | | 0 | 24 | Nested PCR gB |
| | | | | | 0 | 23 | Nested PCR IE1 blood |
| | | | | | 0 | 23 | Nested PCR gB blood |
| 7 | Soroceanu et al. | 2011 | 60 | P | 21 | 26 | IF US28 |
| | | | | | 6 | 26 | IHC US28 |
| | | | | | 18 | 44 | Reverse Transcription PCR US28 |
| 8 | Mitchell et al. | 2008 | 50 | P | 42 | 45 | IHC IE1-72 |
| | | | | | 30 | 33 | IHC pp65 |
| | | | | | 16 | 16 | ISH HCMV DNA (whole DNA) |
| | | | | | 21 | 34 | Real time PCR IE1 (UL123) |
| | | | | | 8 | 34 | Real time PCR gB (UL55) |
| | | | | | 16 | 20 | Nested PCR gB blood |
| 9 | Scheurer et al. | 2008 | 56 | P | 21 | 21 | IHC IE1-72 |
| | | | | | 21 | 21 | ISH HCMV DNA Early |
| 10 | Slinger et al. | 2010 | 59 | P | 20 | 21 | IHC IEA |
| | | | | | 20 | 21 | IHC US 28 |
| 11 | Lucas et al. | 2011 | 46 | P | 25 | 49 | IHC pp65 |
| | | | | | 8 | 49 | IHC IE1 68-72 |
| 12 | Dziurzynski et al. | 2011 | 32 | P | 5 | 5 | Flow cytometry analysis pp65 specific T-cells |
| 13 | Sjöström et al. | 2011 | 58 | P | 53 | 61 | ELISA, IgG |
| 14 | Bhattacharjee * et al. | 2012 | 25 | P | 11 | 11 | Nested PCR DNA |
| | | | | | 6 | 6 | Real time PCR UL123 GBM |
| | | | | | 9 | 12 | WB IE1 |
| | | | | | 9 | 12 | WB pp65 |
| | | | | | 3 | 3 | WB gB |
| 15 | Fonseca et al. | 2012 | 33 | P | 6 | 18 | PCR pp65 |
| 16 | Ranganathan et al. | 2012 | 55 | P | 5 | 5 | PCR gB (UL55) |
| | | | | | 5 | 5 | qPCR gB (UL55) |
| | | | | | 5 | 5 | PCRpp71 (UL82) |
| | | | | | 5 | 5 | qPCRpp71 (UL82) |
| | | | | | 8 | 8 | PCR US28 |
| | | | | | 8 | 8 | qPCR US28 |
| | | | | | 4 | 5 | PCR UL144 |
| | | | | | 4 | 5 | qPCR UL144 |
| | | | | | 9 | 9 | PCR UL17 |
| | | | | | 9 | 9 | qPCR UL17 |
| | | | | | 5 | 5 | PCR UL27 |

| | | | | | | | |
|---|---|---|---|---|---|---|---|
| | | | | | 5 | 5 | qPCR UL27 |
| | | | | | 10 | 10 | PCR UL69 |
| | | | | | 10 | 10 | qPCR UL69 |
| | | | | | 12 | 12 | PCR UL96 |
| | | | | | 12 | 12 | qPCR UL96 |
| | | | | | 4 | 5 | PCR US2 |
| | | | | | 4 | 5 | qPCR US2 |
| | | | | | 9 | 9 | PCR US11 |
| | | | | | 9 | 9 | qPCR US 11 |
| | | | | | 12 | 12 | PCR primers frozen |
| 17 | Ghazi et al. | 2012 | 36 | P | 10 | 11 | IHC IE1 72 |
| | | | | | 5 | 11 | IHC pp65 |
| | | | | | 8 | 9 | ISH CMV DNA |
| | | | | | 11 | 11 | IE specific T-cells in blood |
| | | | | | 11 | 11 | pp65 specific T-cells in blood |
| 18 | Rahbar et al. | 2012 | 54 | P | 79 | 80 | IHC IEA |
| | | | | | 76 | 80 | IHC LA |
| | | | | | 6 | 7 | ISH HCMV DNA IEA LA |
| 19 | Rahbar et al. | 2013 | 52 | P | 74 | 75 | IHC IEA |
| | | | | | 70 | 75 | IHC LA |
| | | | | | 19 | 19 | ISH |
| 20 | Tang et al. | 2013 | 93 | N | 0 | 167 | NGS |
| 21 | Matlaf et al. | 2013 | 48 | P | 5 | 10 | IF pp71 |
| | | | | | 10 | 15 | Rt PCR |
| | | | | | 7 | 10 | Real Time PCR pp71 |
| | | | | | 10 | 10 | qPCR pp71 |
| | | | | | 6 | 10 | ELISA IgG |
| | | | | | 2 | 10 | ELISA IgM |
| | | | | | 9 | 11 | WB pp71 |
| 22 | Stragliotto et al. | 2013 | 19 | P | 42 | 42 | IHC IEA |
| | | | | | 42 | 42 | IHC LA |
| 23 | Ahani et al. | 2013 | 21 | P | 12 | 16 | qPCR |
| 24 | Cosset et al. | 2013 | 72 | N | 0 | 20 | Nested PCR |
| | | | | | 0 | 20 | Nested PCR E1 E2 |
| | | | | | 14 | 31 | ELISA IgG |
| | | | | | 0 | 31 | ELISA IgM |
| | | | | | 0 | 20 | NGS Metagenomic analysis |
| 25 | Garcia-Martinez et al. | 2013 | 74 | N | 1 | 54 | Real Time PCR DNA |
| 26 | Dos Santos et al. | 2014 | 31 | P | 20 | 22 | nPCR gB (UL55) |
| | | | | | 11 | 20 | nPCR gB blood |
| | | | | | 21 | 22 | qPCR pp65 |
| | | | | | 9 | 20 | qPCR pp65 blood |
| 27 | Tang et al. | 2015 | 94 | N | 0 | 34 | NGS DNA |
| 28 | Cimino et al. | 2014 | 71 | N | 0 | 21 | NGS DNA |
| 29 | Ding et al. | 2014 | 29 | P | 16 | 19 | IHC IE1-72 (only GBM) |
| | | | | | 15 | 19 | IHC pp65 (only GBM) |
| | | | | | 12 | 19 | Nested PCR gB (UL55) |
| | | | | | 7 | 19 | Nested PCR gB blood |
| 30 | Yamashita et al. | 2014 | 95 | N | 7 | 10 | IHC IE1/2 |

| | | | | | | | |
|---|---|---|---|---|---|---|---|
| | | | | | 5 | 10 | IHC pp65 |
| | | | | | 10 | 10 | IHC pp28 |
| | | | | | 0 | 10 | FISH DNA |
| | | | | | 0 | 59 | Real Time PCR gB |
| | | | | | 0 | 59 | qPCR IE |
| | | | | | 0 | 5 | WB IE1/2 and pp28 |
| | | | | | 0 | 10 | IHC UL44 |
| 31 | Solomon et al. | 2014 | 89 | N | 0 | 68 | IHC CCH2/DDG9 |
| 32 | Libard et al. | 2014 | 4 | P | 55 | 68 | IHC IE Clone 8B1.2 |
| | | | | | 0 | 68 | IHC IE Clone 6F8.2 |
| | | | | | 55 | 68 | IHC EA Clone QB1/42 |
| | | | | | 55 | 68 | IHC EA Clone BM204 |
| | | | | | 0 | 68 | IHC LA Clone 2D4.2 |
| | | | | | 55 | 68 | IHC LA Clone IG5.2 |
| | | | | | 55 | 68 | IHC pp65 clone 2 and 6 |
| | | | | | 0 | 68 | IHC CCH2+DDG9 |
| 33 | Baumgarten et al. | 2014 | 70 | N | 0 | 91 | IHC IEA monoclonal MCA 2147 |
| | | | | | 0 | 91 | IHC pp65 clone 26 |
| | | | | | 0 | 91 | IHC CCH2/DDG9 |
| | | | | | 0 | 10 | qPCR |
| | | | | | 43 | 67 | Chemiluminescent microparticle immunoassay (CMIA) IgG/IgM) |
| 34 | Priel et al. | 2014 | 86 | N | 0 | 17 | Real Time PCR gB |
| | | | | | 0 | 17 | Real Time PCR UL123 IE1 |
| | | | | | 0 | 19 | Real Time PCR gB blood |
| | | | | | 0 | 19 | qPCR UL123 blood |
| | | | | | 15 | 18 | ELISA IgG |
| | | | | | 0 | 18 | ELISA IgM |
| 35 | Mohammad et al. | 2014 | 51 | P | 5 | 20 | PCR HCMV miRNA UL112 |
| | | | | | 13 | 20 | ELISA IgG |
| | | | | | 1 | 20 | ELISA IgM |
| 36 | Ahani et al. | 2014 | 20 | P | 12 | 16 | Real Time PCR |
| 37 | Shamran et al. | 2015 | 57 | P | 33 | 36 | IHC IE1-72 |
| | | | | | 26 | 36 | IHC LA |
| | | | | | 28 | 36 | IHC pp65 |
| | | | | | 10 | 11 | Nested PCR IE1 |
| | | | | | 10 | 10 | Nested PCR gB |
| | | | | | 10 | 11 | Nested PCR pp65 |
| 38 | Hashida et al. | 2015 | 75 | N | 0 | 39 | qPCR gB |
| | | | | | 0 | 39 | qPCR IE |
| 39 | Leher et al. | 2015 | 79 | N | 0 | 14 | Reverse Transcription PCR blood |
| 40 | Wakefield et al. | 2015 | 63 | P | 14 | 24 | IHC IE1-72 |
| | | | | | 12 | 24 | IHC pp65 |
| | | | | | 13 | 16 | ISH IE pp65 |
| 41 | Sardi et al. | 2015 | 88 | N | 0 | 2 | IHC CCH2/DDG9 (only GBM) |
| | | | | | 0 | 2 | RT PCR DNA |
| | | | | | 2 | 2 | ELISA IgG/IgM |
| 42 | Huang et al. | 2015 | 41 | P | 25 | 25 | IHC IE2-86 (only GBM) |

| | | | | | | | |
|---|---|---|---|---|---|---|---|
| | | | | | 25 | 25 | RT qPCR IE2-86 |
| | | | | | 25 | 25 | WB |
| 43 | Bianchi et al. | 2015 | 26 | P | 30 | 43 | IF IE1 |
| | | | | | 1 | 2 | IF late |
| | | | | | 2 | 6 | IE1-68-72 IF positive |
| | | | | | 17 | 34 | Nested PCR gB(UL55) |
| | | | | | 0 | 8 | pPCR UL55 in blood |
| 44 | Rahbar et al. | 2015 | 53 | P | 100 | 125 | PCR HCMV IE DNA |
| | | | | | 35 | 126 | PCR HCMV IE RNA |
| | | | | | 29 | 42 | ELISA IgG |
| | | | | | 7 | 42 | ELISA IgM |
| | | | | | 10 | 12 | Flow cytometry analysis IE specific T-cells in blood |
| | | | | | 10 | 12 | Flow cytometry analysis pp65 specific T-cells in blood |
| 45 | Dominguez-Valentin et al. | 2016 | 30 | P | 33 | 108 | qPCR pp65 |
| | | | | | 12 | 100 | qPCR pp65 blood |
| | | | | | 18 | 27 | ELISA IgG |
| 46 | Taha et al. | 2016 | 92 | N | 0 | 32 | IHC CCH2/DDG9 |
| | | | | | 0 | 32 | Real Time PCR |
| 47 | Holdhoff et al. | 2016 | 76 | N | 0 | 30 | IHC IEA 86-72 |
| | | | | | 0 | 30 | IHC pp65 |
| | | | | | 0 | 30 | CISH IE1 |
| | | | | | 0 | 30 | CISH pp65 |
| | | | | | 0 | 25 | Real Time PCR DNA US17 |
| | | | | | 4 | 38 | Real Time PCR DNA US17 blood |
| | | | | | 8 | 15 | ELISA IgG |
| 48 | Stangherlin et al. | 2016 | 61 | P | 4 | 10 | IHC HCMV clone CMV 01 (only GBM) |
| | | | | | 2 | 10 | ISH RNA |
| | | | | | 9 | 10 | qPCR pp65 (UL83) |
| 49 | Lin et al. | 2016 | 81 | N | 0 | 45 | ddPCR UL55 |
| 50 | Malekpour et al. | 2016 | 86 | N | 20 | 199 | qPCR |
| 51 | Strong et al. | 2016 | 91 | N | 0 | 323 | NGS RNA |
| 52 | Garcia-Martinez et al. | 2017 | 73 | N | 0 | 110 | IHC CCH2/DDG9 |
| | | | | | 0 | 30 | Nested PCR |
| | | | | | 3 | 122 | RT-qPCR |
| 53 | Johnson et al. | 2017 | 77 | N | 0 | 5 | NGS RNA |
| 54 | Yang et al. | 2017 | 96 | N | 0 | 9 | IHC IE1/2 68-72 |
| | | | | | 0 | 9 | ISH DNA Early |
| | | | | | 0 | 116 | Nested PCR UL144 |
| | | | | | 9 | 116 | qPCR UL73 |
| 55 | Strojnik et al. | 2017 | 90 | N | 0 | 33 | RT/PCR (UL55) |
| 56 | Bahador et al. | 2017 | 24 | P | 40 | 158 | IHC IE1 CCH2/DDG9 |
| | | | | | 65 | 172 | qPCR gB |
| | | | | | 26 | 159 | qPCR pp65 |
| | | | | | 18 | 130 | qPCR gB blood |
| | | | | | 12 | 119 | qPCR pp65 blood |
| | | | | | 38 | 48 | ELISA IgG |

| | | | | | | | |
|---|---|---|---|---|---|---|---|
| 57 | Basset et al. | 2017 | 69 | N | 1 | 21 | IHC IE1 |
| | | | | | 7 | 21 | IHC pp65 |
| 58 | Han et al. | 2018 | 39 | P | 33 | 68 | IF gB |
| | | | | | 29 | 68 | IF gH |
| 59 | Han et al. | 2017 | 38 | P | 78 | 95 | IHC IE1-72 (only GBM) |
| | | | | | 65 | 95 | IHC pp65 clones 2 and 6 |
| | | | | | 30 | 95 | Nested PCR HCMV DNA |
| | | | | | 65 | 95 | ELISA IgG |
| | | | | | 19 | 95 | ELISA IgM |
| 60 | Hu et al. | 2017 | 40 | P | 25 | 25 | IHC IE86 |
| 61 | Liang et al. | 2017 | 43 | P | 23 | 23 | IHC IE86 |
| 62 | Zavala-Vega et al. | 2017 | 67 | P | 0 | 7 | IHC IE1 |
| | | | | | 7 | 7 | Nested PCR IE1 |
| | | | | | 5 | 7 | Semi nested PCR IE1 |
| | | | | | 3 | 7 | PCR gB |
| 63 | Xing et al. | 2016 | 66 | P | 40 | 43 | IHC pp65 |
| | | | | | 28 | 43 | ISH DNA pp65 |
| 64 | Chen et al. | 2017 | 27 | P | 11 | 14 | IHC IE1 |
| | | | | | 11 | 14 | IHC pp65 |
| | | | | | 9 | 14 | Nested PCR UL55 |
| | | | | | 13 | 14 | ELISA IgG |
| | | | | | 2 | 14 | ELISA IgM |
| 65 | Ahn et al. | 2018 | 22 | P | 0 | 20 | RT PCR DNA |
| | | | | | 20 | 20 | ELISA IgG |
| | | | | | 0 | 20 | ELISA IgM |
| 66 | Al-Sherify et al. | 2019 | 23 | P | 8 | 14 | Nested PCR gB |
| 67 | Deshpande et al. | 2018 | 28 | P | 7 | 7 | RT PCR RNA UL112-3p |
| 68 | Meng et al. | 2018 | 49 | P | 21 | 21 | Flow cytometry analysis pp65 specific T-cells in blood |
| 69 | Liu et al. | 2018 | 45 | P | 107 | 151 | Flow cytometry analysis pp65 specific T-cells in blood |
| 70 | Adnan et al. | 2019 | 68 | N | 1 | 112 | Nested PCR gB |
| 71 | Foster et al. | 2019 | 34 | P | 97 | 188 | ELISA IgG |
| 72 | Loit et al. | 2019 | 82 | N | 0 | 42 | IHC IE1 |
| | | | | | 1 | 28 | qPCR tissue |
| | | | | | 6 | 31 | PCR blood |
| | | | | | 32 | 47 | ELISA IgG |
| | | | | | 2 | 47 | ELISA IgM |
| 73 | Nikolova et al. | 2019 | 84 | N | 1 | 10 | PCR tissue |
| | | | | | 0 | 10 | PCR blood |
| 74 | Frey et al. | 2020 | 35 | P | 12 | 44 | HCMV IgG |
| 75 | Goerig et al. | 2020 | 37 | P | 12 | 44 | RT PCR DNA |
| | | | | | 23 | 44 | ELISA IgG |
| 76 | Idriss et al. | 2020 | 42 | P | 1 | 9 | Nested PCR (GBM) |
| 77 | Liman et al. | 2020 | 44 | P | 0 | 82 | IHC IE1 8B1.2 |
| | | | | | 0 | 82 | IHC LA 2D4.2 |
| | | | | | 0 | 82 | IHC LA 1G5.2 |
| | | | | | 0 | 82 | ISH RNA |
| | | | | | 55 | 82 | qPCR |

| | | | | | | | |
|---|---|---|---|---|---|---|---|
| 78 | Maleki et al. | 2020 | 47 | P | 16 | 45 | IHC pp65 clone 2 and 6 |
| | | | | | 44 | 45 | RT PCR pp65 |
| 79 | Weathers et al. | 2020 | 64 | P | 61 | 65 | pp65 T-cells blood |
| 80 | Yuan et al. | 2020 | 97 | N | 0 | 111 | NGS RNA |
| 81 | Ursu et al. | 2021 | 62 | P | 16 | 69 | RT PCR pp65 |
| | | | | | 69 | 103 | ELISA IgG |

HCMV; human cytomegalovirus; n; number, P; positive, N; negative, ELISA; RT; Reverse Transcription; IHC; immunohistochemistry; NGS; Next-Generation Sequencing; ISH; in situ hybridisation.
